# Anti-hyperglycaemic activity of *swietenia macrophylla* king (meliaceae) seed extracts in normoglycaemic rats undergoing glucose tolerance tests

**DOI:** 10.1186/1749-8546-8-11

**Published:** 2013-05-17

**Authors:** Mohd Akmal Hashim, Mun Fei Yam, Sook Yee Hor, Chung Pin Lim, Mohd Zaini Asmawi, Amirin Sadikun

**Affiliations:** 1 School of Pharmaceutical Sciences, Universiti Sains Malaysia, 11800 Pulau Pinang, Malaysia; 2 Institute of Marine Biotechnology, Universiti Malaysia Terengganu, 21300 Terengganu, Malaysia

## Abstract

**Background:**

*Swietenia macrophylla* King (Meliaceae) is used to treat diabetes mellitus in Malaysia. This study aims to evaluate the anti-hyperglycaemic potential of petroleum ether (PE), chloroform (CE) and methanol (ME) extracts of *S. macrophylla* seeds, in normoglycaemic and streptozotocin (STZ)-induced diabetic rats.

**Methods:**

Following treatment of normoglycaemic rats with *S. macrophylla* seed extracts, hypoglycaemic and intraperitoneal glucose tolerance tests (IPGTT) were performed, and blood glucose concentrations were measured. Similarly, glucose concentrations were measured after 1 and 14 days of extract treatment of STZ-induced diabetic rats. Glucose absorption by isolated everted intestine and glucose uptake by isolated abdominal muscle were tested after treatment with seed extracts. Gas chromatography mass spectrometry (GC-MS) analysis was performed on PE of *S. macrophylla* seeds to identify the compounds responsible for its activity.

**Results:**

None of the extracts had a significant effect on the blood glucose levels of 60 randomly selected normoglycaemic (normal) and diabetic rats undergoing hypoglycaemic tests. PE, however, significantly reduced blood glucose levels in 30 randomly selected normoglycaemic rats undergoing IPGTT tests 30–120 minutes after glucose administration. Repeated doses of 1000 mg/kg and 500 mg/kg PE to STZ-induced diabetic rats for 14 days did not reduce blood glucose levels significantly. PE did not significantly reduced the intestinal absorption of glucose, but significantly increased glucose uptake by abdominal muscle in the absence or presence of insulin. GC-MS analysis indicated that diterpenes, triterpenoids, fatty acid methyl esters, aldehydes and phytosterols may be responsible for the glucose lowering effects of PE.

**Conclusion:**

PE extracts of *S. macrophylla* seeds showed anti-hyperglycaemic activity on IPGTTs . GC-MS analysis on the PE revealed that several compounds, including fucosterol and β-sitosterol, may be responsible for these anti-hyperglycaemic properties.

## Background

Diabetes mellitus is a disease in which the homeostasis of carbohydrate, protein and lipid metabolism is improperly regulated by insulin, resulting in elevated fasting and post-prandial blood glucose concentrations. Chronic hyperglycaemia causes many complications, including nephropathy, retinopathy, neuropathy, and macrovascular and microvascular damage [1]. Its symptoms include polyurea, polydipsia, polyphagia, weight loss, fatigue, cramps, constipation and blurred vision. In 2004, the World Health Organization (WHO) estimated that the prevalence of diabetes worldwide will increase from 171 million in 2000 to 366 million in 2030 [2]. The Malaysia Diabetes Association has estimated that approximately 1.7 million people are currently affected and that further industrialisation and modernization may result in a double of this number by 2030 [3].

Generally, patients with diabetes mellitus are treated with oral hypoglycaemic agents (OHA) and insulin [4]. These drugs, however, are not suitable for use during pregnancy and can produce serious side effects [5-8]. The use of medicinal plants to treat diabetes mellitus is popular, as herbal drugs are generally regarded as free of toxic effects [9]. Therefore, the search for more effective and safer herbal anti-diabetic agents has become an area of active research.

*Swietenia macrophylla* King (Meliaceae), commonly known as big leaf mahogany (vernacular) and ‘skyfruit’ (local), is used to treat diabetes and high blood pressure in Malaysia [10]. *S. macrophylla* seeds have been reported to have anti-inflammatory, anti-mutagenic and anti-tumor activities [11] and to be effective against diabetes in rats [12]. In Chinese pharmacology and other traditional medicines, this plant has antipyretic, antifungal, and antihypertensive properties, pharmacological effects obtained from dried seeds, finely ground to powder [13].

Traditionally, raw seeds of *S. macrophylla* are chewed to treat diabetes. In Malaysia, these seeds are chewed or pounded and swallowed to treat high blood pressure [10] and in India, they are used to treat diabetes and hypertension [14]. We therefore elected to extract the seeds using the maceration method rather than the soxhlet method since the former method exposes the seeds to lower temperatures. The soxhlet method was avoided since prolonged heating may degrade thermolabile compounds [15].

This study was designed to investigate *S. macrophylla* seed extracts in different *in vivo* and *in vitro* diabetic models in order to evaluate their anti-hyperglycaemic properties and to elucidate the possible mechanism underlying these properties. Compounds possibly responsible for these activities were determined by GC-MS analysis.

## Materials and methods

### Chemicals and reagents

All chemicals and solvents were of analytical grade. Petroleum-ether (60–80°C), chloroform and methanol were purchased from Merck (Darmstadt, Germany). Streptozotocin (STZ) was purchased from Sigma Chemicals (St. Louis, MO, USA).

### Plant materials

The fruit seeds of *S. macrophylla* were collected from the area of Jitra, Malaysia, between December 2008 and February 2009 and identified by Mr. Vellosamy Shunmugam, a plant taxonomist from the School of Biological Sciences, Universiti Sains Malaysia (USM). A voucher specimen was deposited (11239) in the herbarium of the School of Biological Sciences, USM.

### Extraction of plant material

The fruits were peeled to get the seeds. The seeds were dried in an oven at 45°C for one week, then ground to a coarse powder in an electrical grinder, weighed and stored in a dry place. The dried powder (2.2 kg) was continuously extracted by the maceration method [16] using petroleum ether (60–80°C), chloroform (CE) or methanol (ME), three times each.

The solvents from each extract were removed using a rotary evaporator, and the extracts were stored at -70°C for 48 hours, and freeze dried under vacuum at -40°C for 24 h using a freeze-dryer (Labconco Corporation, Denmark). Each dried extract was kept in tightly covered glass bottles and stored at 4°C. The yields of each extract are shown in Table 1.

**Table 1:** **Yields of extracts of *S. macrophylla *seeds**

**Method of extraction**	***S. macrophylla *(gram)**	**Solvent**	**Extracts (gram)**	**Yield (%)**
		Petroleum ether	189.2	8.6
	2200	Chloroform	136.4	6.2
Maceration		Methanol	118.8	5.4

### Experimental animals

Randomly selected male Sprague–Dawley rats (200–250 g; 12–16 weeks of age) were obtained from the animal house of the School of Pharmaceutical Sciences, USM and kept under standard conditions of 12 h: 12 h light and dark in polypropylene cages and fed a standard laboratory diet and water ad libitum. All experiments included six rats per group, and all experiments adhered to the principles of Laboratory Animal Care and the protocols of the Animal Ethics Committee of USM (USM/PPSF/50 (081) Jld 2.

### Hypoglycaemic test in normal rats

Thirty randomly selected normal male Sprague–Dawley rats weighing 200–250 g were arbitrarily divided into five groups of 6 rats each. After an overnight (16 h) fast, Group 1 (negative control) rats were treated with 4% Tween 80 (10 mL/kg), group 2 (positive control) rats with glibenclamide (10 mg/kg), group 3 rats with 1000 mg/kg petroleum ether (PE), group 4 with 1000 mg/kg CE, and group 5 with 1000 mg/kg ME, all by gavage. Blood samples were drawn from each rat’s tail vein at just prior to treatment and 1, 2, 3, 5 and 7 h after treatment, and blood glucose concentrations were determined using an Accu-Check Advantage II Clinical Glucose meter (Roche, USA).

### Intraperitoneal glucose tolerance test (IPGTT) in normal rats

Thirty rats were divided into five groups of 6 rats each. After an overnight (16 h fast), the rats were treated as described above. Sixty minutes later, each rat was administered glucose (500 mg/kg b.w.) by intraperitoneal (i.p.) injection. Blood samples were drawn from each rat’s tail vein at -60 min (just before gavage), 0 min (before glucose load), and 15, 30, 45, 60, 90 and 120 min after glucose administration. Blood glucose concentrations were determined as above.

### Induction of diabetes in normal rats

Diabetes was induced in overnight fasted (16 h) Sprague–Dawley rats by a single i.p. injection of 60 mg/kg streptozotocin (STZ) dissolved in normal saline (0.9% NaCl, pH 4.5) just prior to administration [17]. Hyperglycaemia was confirmed by elevations in blood glucose concentrations 72 h later and by polyurea and loss of body weight. Rats with fasting blood glucose (FBG) concentrations above 15 mmol/L were considered diabetic.

### Hypoglycaemic tests in diabetic rats

Thirty diabetic rats weighing 170–220 g were arbitrarily divided into five groups of 6 rats each, fasted overnight, and treated as above. Blood samples were drawn at 0 h (just before gavage), and 1, 2, 3, 5 and 7 h after treatment. Blood glucose concentration was measured as above.

### Treatment of diabetic rats for 14 days

Twenty male rats with STZ-induced diabetes were randomly divided into four groups of 5 rats each. Group 1 (negative control) rats were treated with 4% Tween 80 (10 mL/kg), group 2 (positive control) rats with metformin (500 mg/kg), group 3 rats with 1000 mg/kg PE and group 4 rats with 500 mg/kg PE, all by gavage, twice daily (at 9 a.m. and 9 p.m.) for 14 days. Body weight was measured every two days. Fasting blood glucose levels and body weight of the rats were determined before and after the 14-day treatment period.

### *In vitro* glucose absorption by isolated everted intestine

**Figure 1:**
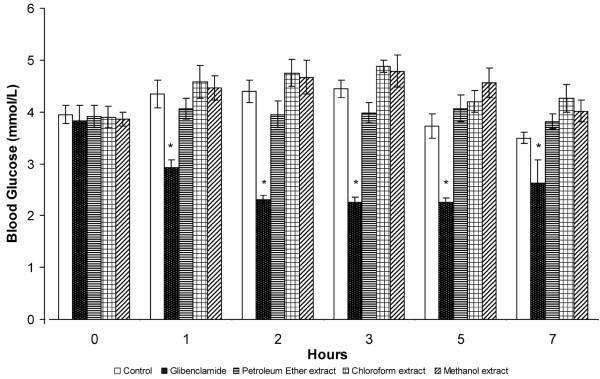
**Effect of orally administered *S. macrophylla *seed extracts (1 g/kg b.w.) on hypoglycaemic tests in normal rats. **Each value represents the mean ± SD for six rats. * *P *< 0.05 compared with negative control rats.

**Figure 2:**
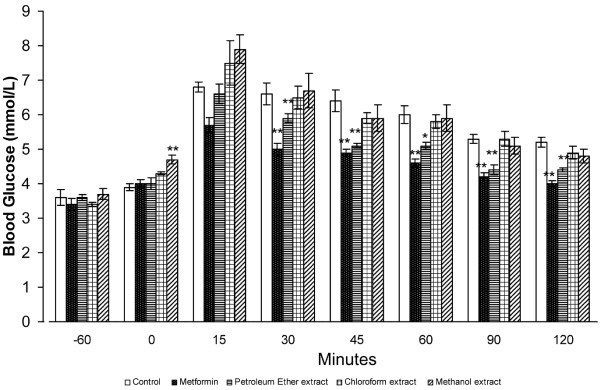
**Effect of orally administered *S. macrophylla *seed extracts (1 g/kg b.w.) on IPGTT results in normal rats. **Each value represents the mean ± SD for six rats. * *P *< 0.05, ** *P *< 0.005 compared with negative control rats.

Although none of the three extracts of *S. macrophylla* seeds, PE, CE, and ME, had any hypoglycaemic activity in normal rats (Figure 1), PE showed the highest anti-hyperglycaemic activity in the IPGTT test (Figure 2). Therefore, the anti-hyperglycaemic properties were further investigated.

Following an overnight fast, male Sprague–Dawley rats weighing 250–300 g were sacrificed by a blow to the head, followed by cervical dislocation and dissection of their abdominal walls. The effect of PE on glucose absorption by the rat intestine was assessed as described [18]. After the abdomen was opened, the small intestine was excised, placed in oxygenated Tyrode’s solution (342 mM NaCl, 6.7 mM KCl, 5.9 mM CaCl_2_.2H_2_O, 5.3 mM MgCl_2_, 59.5 mM NaHCO_3_, 2.08 mM NaH_2_PO_2_, 5.5 mM glucose) and rinsed with the same solution to remove intestinal contents. The jejunum (24- to 50-cm from the pylorus) was identified and isolated from the small intestine, everted and cut into 5-cm long pieces. One end of each piece was tied with a cotton thread, and filled with 1 mL of Tyrode’s solution, and the other end was tied, forming a sac. Sacs were incubated in 15 mL Tyrode’s solution for 90 min in the presence of 1 and 2 mg/mL PE, 1 and 2 mg/mL acarbose, an α-glucosidase inhibitor as positive controls, or Tyrode’s solution alone as the negative control. At the end of the incubation period, the glucose concentrations inside the sacs and in the incubation media were determined by the Statfax machine (model 1906, 45 Watts, Awareness Tech, Inc. Palm City, FL, USA) so that the amount of glucose uptake could be calculated. Results were expressed as mg glucose absorption or glucose uptake per g tissue weight.

### *In vitro* glucose uptake by isolated abdominal muscles

Glucose uptake by rat abdominal muscles was assessed as described [19], with some modifications. Male Sprague–Dawley rats weighing 200-250 g were sacrificed as above and skinned at the abdomen to expose their abdominal muscles. The rectus abdominus was removed from the abdominal muscle, and the clean muscle was transferred to Krebs solution aerated with 95% O_2_. Both sides of the abdominal muscles were cut into small squares weighing approximately 150–300 mg, and kept in Krebs-Ringer bicarbonate buffer (KRB; 118 mM NaCl, 5 mM KCl, 1.28 mM CaCl_2_, 1.2 mM KH_2_PO_4_, 1.2 mM MgSO_4_, 25 mM NaHCO_3_) for 10 min at 37°C in the presence of 95% O_2_ and 5% CO_2_. The solution was replaced by Krebs solution containing 11.1 mM glucose and samples were collected for baseline readings. Tissue samples were incubated with 1 and 2 mg/mL PE or 1 mg/mL metformin in the presence or absence of insulin (1 I.U./mL). The solutions were aerated for 5 min and incubated for 30 min at 37°C while shaking at 96 rpm. Muscle weight was measured to determine the amount of glucose uptake per gram, and glucose concentration was measured using the Statfax machine.

### Gas chromatography–mass spectrometry (GC-MS) analysis

GC-MS analysis of the PE extract was performed using an Agilent 6890 gas chromatography instrument coupled to an Agilent 5973 mass spectrometer and Agilent Chemstation software (Agilent Technologies, Palo Alto, CA, USA). Compounds were separated on a HP-5MS, 30 m × 0.25 mm i.d. capillary column coated with 0.25-μm film. The oven temperature was held at 70°C for 2 min and increased to 285°C isothermally over 20 min. Helium was the carrier gas at a flow rate of 1.2 mL/min. The injector and detector temperatures were 280°C and 250°C, respectively. The parameters of the HP 5973 mass detector were: ion mass/charge ratio, 20–500 m/z; scan mode and ionisation energy of 70 eV. Sample components were identified by matching their mass spectra with those recorded in the NIST/Wiley Library and comparing the literature data with GC retention indices [20].

### Statistical analysis

All data are expressed as mean ± standard deviation (SD), and all statistical analyses were performed using SPSS ver 11.5 software (SPSS Inc., Chicago, IL). Mean between group differences were analysed by one-way ANOVA at an α-value (probability level) *P* < 0.05 followed by Dunnett *t*-test as a post-*hoc* test, except for the 14-day treatment where the means differences between pre and post-treatment parameters were analysed by paired *t*-tests and considered significant at *P* < 0.05.

## Results

### Hypoglycaemic test in normal rats

Oral administration of 1000 mg/kg of the three *S. macrophylla* seed extracts to normal rats, had no effect on fasting blood glucose concentrations, compared with control rats, whereas the positive control, 10 mg/kg glibenclamide, significantly reduced fasting blood glucose concentrations (Figure 1).

### IPGTT in normal rats

Figure 2 shows that the blood glucose levels in each group increased after i.p. loading of 500 mg/kg glucose. Although ME had no effect on blood glucose concentrations, PE significantly reduced blood glucose at 30–120 min after glucose loading. Similarly, the positive control, metformin, also significantly reduced the blood glucose concentrations.

### Hypoglycaemic test in diabetic rats

**Figure 3:**
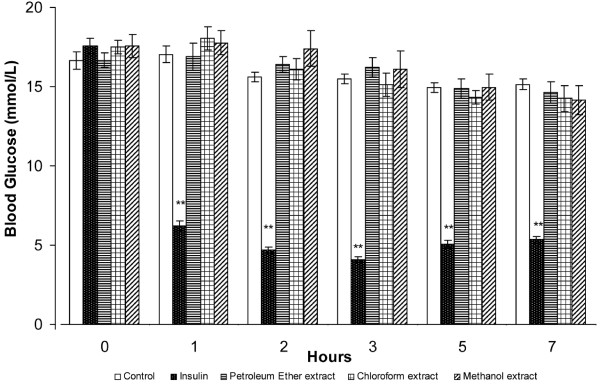
**Effect of orally administered *S. macrophylla *seeds extracts (1 g/kg b.w) on hypoglycaemic tests in STZ-induced diabetic rats.** Each value represents the mean ± SD for six rats. ** *P *< 0.001 compared with negative control rats.

Oral administration of 1000 mg/kg PE, CE and ME to diabetic rats (Figure 3) did not significantly affect their fasting blood glucose concentrations compared with control rats. Treatment with 5 IU/kg insulin, however, significantly reduced fasting blood glucose concentrations.

### Effects of treatment for 14 days

**Figure 4:**
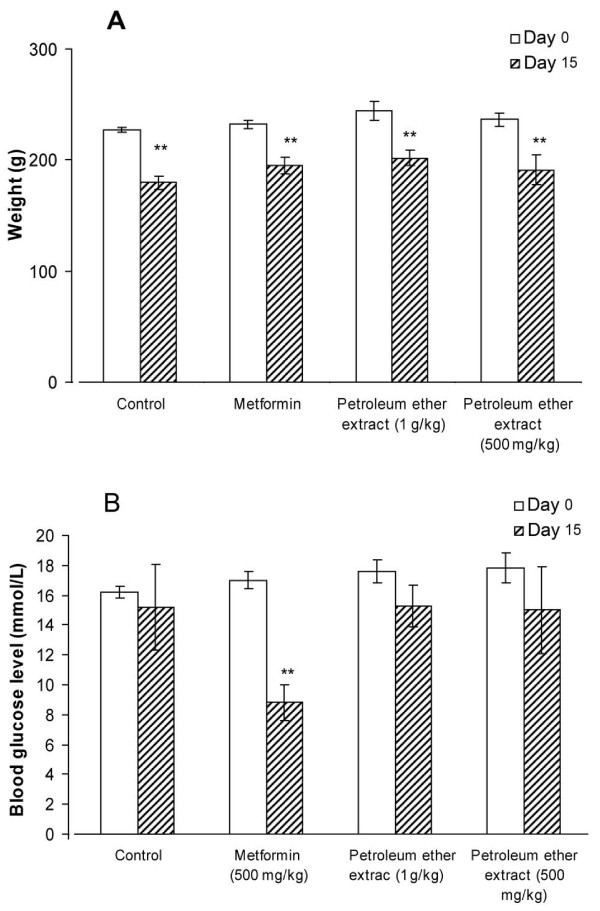
**Effects on body weight (A) and blood glucose concentration (B) after 14 days of oral administration of 10 mL/kg 4% Tween 80 (negative control), 500 mg/kg metformin (positive control), and 500 mg/kg and 1000 mg/kg PE to diabetic rats. **Each value represents the mean ± SD for five rats. **P < 0.01 compared with negative control rats.

Treatment with 4% Tween 80 for 14 days significantly reduced the body weight (*P* = 0.008) of rats with STZ-induced diabetic rats, as did 14 days of treatment with 500 mg/kg metformin (*P* = 0.006) or 500 (*P* = 0.008) or 1000 (*P* = 0.006) mg/kg PE (Figure 4A).

Treatment with 4% Tween 80 or 500 or 1000 mg/kg PE for 14 days had no effect on blood glucose concentrations, whereas 500 mg/kg metformin significantly (*P *= 0.007) reduced blood glucose levels (Figure 4B).

## *In vitro* experiments

### Effect of PE on glucose absorption by isolated everted intestine

**Figure 5:**
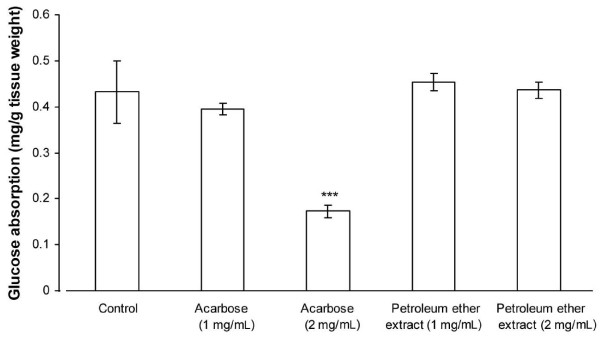
**Effects of PE and acarbose, at concentrations of 1 and 2 mg/mL, on glucose absorption by everted intestine. **Each value represents the mean ± SD of 8 samples. *** *P* < 0.01 compared with the control group.

Acarbose (1 mg/mL) and PE (1 and 2 mg/mL) did not significantly inhibit glucose absorption through everted intestinal sacs, whereas 2 mg/mL acarbose had a significant (*P* = 0.007) effect (Figure 5).

### Effect of PE on glucose uptake by isolated abdominal muscle

**Figure 6:**
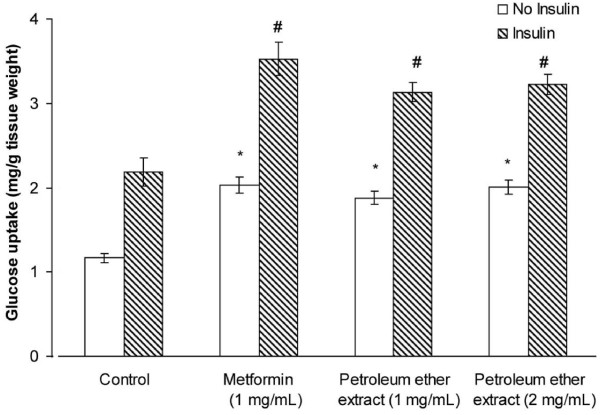
**Effects of PE (1 and 2 mg/mL) and metformin (1 mg/mL) on glucose uptake by isolated rat abdominal muscles in the presence or absence of insulin. **Each value represents the mean ± SD of 8 samples. * *P*< 0.05 compared with control in the presence of insulin; # *P*< 0.05 compared with control in the absence of insulin.

In the absence of insulin, 1 mg/mL metformin (*P* = 0.042) and 1 (*P* = 0.034) and 2 (*P* = 0.048) mg/mL PE significantly increased glucose uptake by abdominal muscles (Figure 6). These effects were further elevated in the presence of 1 IU/mL insulin, with 1 mg/mL metformin (*P* = 0.003) and 1 (*P* = 0.009) and 2 (*P* = 0.005) mg/mL PE significantly increasing glucose uptake compared with control.

### GC-MS analysis of PE

**Figure 7:**
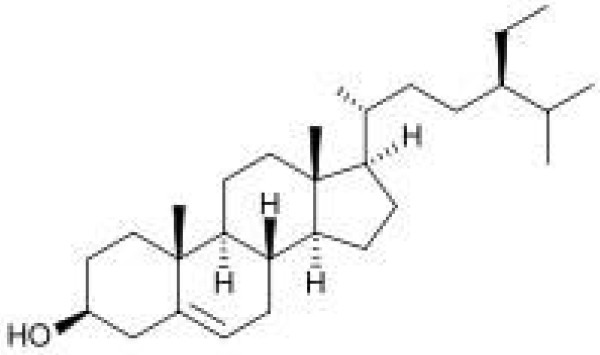
The chemical structure of β-sitosterol.

**Figure 8:**
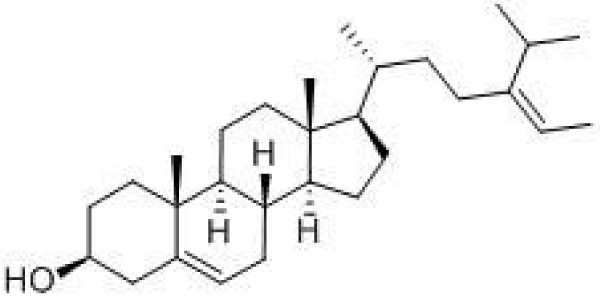
The chemical structure of fucosterol.

GC-MS analysis identified the constituents of PE (Table 2) as hexadecanoic acid methyl ester, n-hexadecanoic acid, 9-octadecenoic acid (Z)-methyl ester, linoleic acid, gamma-tocopherol, fucosterol and β-sitosterol. The major constituents were linoleic acid (72.46%), n-hexadecanoic acid (16.44%), 9-octadecenoic acid (Z)-methyl ester (3.42%), β-sitosterol (0.41%) [Figure 7] and fucosterol (2.45%) [Figure 8].

**Table 2:** **The primary compounds in PE of *S. macrophylla *seeds**

**Retention time (minute)**	**Compounds**	**Peak area (%)**	**Molecular formula**
10.31	Hexadecanoic acid, methyl ester	0.17	C17H34O2
10.69	n-Hexadecanoic acid	16.44	C16H32O2
11.17	9-Octadecenoic acid (Z)- methyl ester	3.42	C19H36O2
11.79	9,12-Octadecadienoic acid (Z,Z)-Linoleic acid	72.46	C18H32O2
16.44	Gamma-tocopherol	0.38	C28H48O2
20.01	Fucosterol	2.45	C29H48O
20.20	β- sitosterol	0.41	C29H50O

## Discussions

Diabetes mellitus is a metabolic disorder involving oxidative stress, which induces insulin resistance in the peripheral tissues and impairs insulin secretion by pancreatic β-cells [21-23]. A large number of hypoglycaemic/antidiabetic plants and herbs have been studied by modern methods and are starting to be introduced into modern therapy.

We utilized the IPGTT rather than the oral glucose tolerance test (OGTT) to investigate the anti-hyperglycaemic properties of *S. macrophylla* seed extracts. The OGTT can yield false positive results due to the sticky or viscous nature of plant extracts. These extracts may interfere with glucose absorption in the gastrointestinal tract when glucose is loaded one hour after administration of the extract. We therefore decided to deliver the extract/drug orally but to load glucose intraperitoneally one hour.

We found that PE, at a dose of 1000 mg/kg b.w., reduced blood glucose concentration in normal rats, suggesting that PE has anti-hyperglycaemic properties, as well as significantly suppressing the increase in blood glucose levels following glucose loading. The anti-hyperglycaemic potential of PE was further evaluated using isolated everted jejunal sacs and isolated abdominal muscles. Blood glucose concentrations in everted jejunal sacs are reduced by inhibition of glucose absorption, for example by α-glucosidase inhibitors such as acarbose [24]. We found that 2 mg/mL acarbose but not 1.0 or 2.0 mg/mL PE significantly inhibited intestinal glucose absorption. By contrast, both concentrations of PE significantly increased glucose uptake by isolated abdominal muscle in the absence or presence of insulin. Therefore, although PE may not inhibit glucose absorption by the rat small intestine, it may promote glucose utilisation by muscle tissue.

PE may significantly reduce fasting blood glucose concentration in normal rats by other mechanisms of action, including the stimulation of the residual pancreatic mechanism or by increasing the peripheral utilisation of glucose [25]. Moreover, the anti-hyperglycaemic properties of PE may be associated with more than one mechanism, including the modulation of insulin secretion or action, extrapancreatic and pancreatic effects [26-28], antioxidant activity [29], enhancement of β-cell glucose metabolism or activation of enzyme systems that generate cyclic AMP or phospholipid derived messengers [30].

STZ-induced hyperglycaemia is a useful experimental model for studying the activity of hypoglycaemic agents [31]. STZ selectively destroys pancreatic insulin secreting β-cells. Using this model, we assessed whether 14 days of treatment with PE could induce significant recovery of these diabetic rats. We found that neither 500 nor 1000 mg/kg PE twice daily for 14 days significantly affected blood glucose concentrations in these diabetic rats.

GC-MS analysis of PE showed the presence of diterpene and triterpenoids, fatty acid methyl esters, aldehydes and phytosterols. The high concentrations of fatty acids and sterol compounds may be responsible for the antihyperglycaemic activity of PE. In addition, significantly reduced blood glucose concentrations have been reported in rats fed fucosterol [32], suggesting that *Pelvetia siliquosa* fucosterol may have a significant antihyperglycaemic effect in diabetic rats. Therefore, fucosterol may also be important in the anti-hyperglycaemic effects of PE of *S. macrophylla* seeds.

## Conclusion

PE from *S. macrophylla* seeds was found to possess anti-hyperglycaemic activity on the IPGTT. GC-MS analysis of the PE revealed several compounds, including fucosterol and β-sitosterol, that may be responsible for the anti-hyperglycaemic properties of this extract.

## Abbreviations

PE: Petroleum-ether extract; CE: Chloroform extract; ME: Methanol extract; IPGTT: Intraperitoneal glucose tolerance test; STZ: Streptozotocin; g/b.w: Gram per body weight; GC-MS: Gas chromatography–mass spectrometry; IU: International unit.

## Competing interests

The authors declare that they have no competing interests.

## Authors’ contributions

MAH, MFY, MZA and AS designed the study. MAH, MFY, SYH and CPL performed the experiments. MAH and MFY wrote the manuscript. All the authors have read and approved the final manuscript.
